# Clinical baseline and prognostic difference of platelet lymphocyte ratio (PLR) in right-sided and let-sided colon cancers

**DOI:** 10.1186/s12885-017-3862-8

**Published:** 2017-12-20

**Authors:** Lin Yang, Wenzhuo He, Pengfei Kong, Chang Jiang, Qiong Yang, Qiankun Xie, Liang Ping Xia

**Affiliations:** 1 Sun Yat-sen University cancer center, 651 Dongfeng Road east, Guangzhou, 510060 China; 2 State Key Laboratory of Oncology in Southern China, Guangzhou, China; 3 Collaborative Innovation Center for Cancer Medicine, Guangzhou, China; 4 The Sun-yat sen memorial hospital, Guangzhou, China

**Keywords:** Left-sided colon cancer (LCC), Right-sided Colon Cancer (RCC), Platelet lymphocyte ratio (PLR), Overall survival (OS), Prognostic difference

## Abstract

**Background:**

Right-sided colon cancer (RCC) and left-sided colon cancer (LCC) differ with respect to their biology and genomic patterns, but inflammatory index variation did not fully investigate. This study aimed to examine the difference of inflammatory indexes and its value between RCC and LCC.

**Methods:**

The differences of common clinicopathologic factors, inflammatory indexes including PLR (Platelet lymphocyte ratio) between LCC and RCC were analyzed in the training cohort with logistic regression model, subsequently, confirmed in validation cohort. Kaplan-Meier analysis was applied for the analysis of the survival difference distinguished by the PLR and the Nonparametric Test was adopted to demonstrate the difference of PLR variation with the standard TNM classification between RCC and LCC.

**Results:**

A total of 1846 CRC patients entered the study, 744 (40.3%) patients were RCC, 1102 (59.7%) were LCC. The patients’ number in both cohorts was 923. It was found that LCC patients in the training cohort significantly to be with higher CEA, adenocarcinoma, early UICC/AJCC stage, p-MMR (mismatch-repair proficient), and lower PLR, and the later four features were confirm in validation cohort. Higher PLR, the unique inflammatory index, was significantly associated with poorer OS in LCC cohort (*P* = 0.002) and was elevated with the TNM stage in the LCC patients (*P* < 0.001), however, the two relationships did not sustain in RCC patients.

**Conclusion:**

Expect the classical characteristics, PLR, an inexpensive and easily assessable inflammatory index was found first time to be significant differ between LCC and RCC. Further, elevated PLR associated with poor OS (overall survival) in the LCC and more common in advanced TNM stage.

**Electronic supplementary material:**

The online version of this article (10.1186/s12885-017-3862-8) contains supplementary material, which is available to authorized users.

## Background

Colon cancer (CRC) has always be viewed as two different subtype since *Bufill* et al. firstly observed the clinical characteristics difference between right-sided colon cancer (RCC) and left-sided colon cancer (LCC) [1].Since then, not only the classical characteristics differences such as RCC tend to have more proportion of anemia, intestinal perforation, mucinous histology type, higher CEA (carcino-embryonic antigen) level, younger female, etc. were proven in numerous studies, but also, the molecular features were found to be different between the two subtypes, for example, CpG island methylation, d-MMR(mismatch repair deficiency), KRAS mutation, EGFR A13 loss, BRAF mutation, etc. was more commoner in RCC [2, 3]. Recently, the difference of the two subtypes attract more interest because of their different reaction to targeted agents. *Gibbs* et al. has reported that in the patients received the Bevacizumab, the RCC patients has the most obvious PFS (progression-free survival) benefit [4]. However, the results of the *Boisen* revealed that there exists the apparent survival advantage in the LCC when combined the chemotherapy with the Bevacizumab [5]. Furthermore, in the metastasis-CRC in China and KRAS-wide patients, the combined cetuximab and chemotherapy could enhance the ORR (objective response rate), PFS (Progression-free survival) and OS (overall survival) in LCC patients without the survival benefit in the RCC [6, 7].

In the exploratory classification system of consensus molecular subtypes (CMS), CRC can be divided into four types [8–10]: CMS1 (MSI Immune, 14%), CMS2 (Canonical, 37%), CMS3 (Metabolic, 13%), and CMS4 (Mesenchymal, 23%), RCC and LCC also show different features. RCC relate to CMS2, with the features of SCNA high, microsatellite stable, weak immune activation, which might more insensitive to immunotherapy [11]. The phase II clinical trial has demonstrated that only the mismatch repair–deficient (d-MMR) subset of CRC to be a good candidate for the PD-1 blockage immunotherapy [12]. An estimated 20–25% of RCC stage II cancers being MSI-high (microsatellite instability-high) compared with the rare existence in LCC across all stages [8–10, 13–15], this partially explains the lower immunogenicity in LCC. In fact, the exact mechanism why lower immunogenicity in LCC which relate to CMS2 and its better outcome with the targeted therapy remained unknown [4, 7, 11, 16]. Recently, *Asaf* et al. has found that Ly6G + neutrophils suppress intraluminal natural killer cell (NK)-mediated tumor cell clearance and facilitate extravasation of carcinoma cells [17], it indicate that inflammatory response may inhibit immune response. Does this correlation can help to explain the above mechanism? But the data of the difference of inflammatory parameters between RCC and LCC is rare. Though, some systematic inflammatory biomarkers such as the prognostic Nutritional Index (PNI), Glasgow prognostic score (mGPS), neutrophil lymphocyte ratio (NLR), and platelet lymphocyte ratio (PLR), have been shown to have prognostic value in various tumors, including CRC [18–20]. The prognostic value for CRC had been shown in the CRC, but not the Asians and it had not point out the prognostic difference in the LCC and RCC [21].

## Method

A total of 1846 eligible colorectal cancer patients treated at Sun Yat-sen University Cancer Center between December 2003 and August 2015 were retrospectively enrolled after the exclusion criteria of patients without complete follow-up data. The inclusion criteria for the study are as follows: (i) pathological evidence of adenocarcinoma of CRC; (ii) complete baseline clinical information and laboratory data; and (iii) complete follow-up data. Treatment regimen is implemented based on the NCCN guidelines https://www.nccn.org/. Simply, stage I colon cancer receive radical surgery and colon cancer patients with low-risk stage II disease can be enrolled in a clinical trial, observed without adjuvant therapy, or considered for capecitabine or 5-FU/leucovorin(LV). For patients with high-risk stage II disease, they can be considered for adjuvant chemotherapy with 5-FU/LV (5-Fluorouracil/Leucovorin), capecitabine, FOLFOX (5- Fluorouracil+oxaliplatin+Leucovorin), CapeOX (Oxaliplatin+ Capecitabine), FLOX, or observation. Radiotherapy, chemotherapy and surgery were combined for the treatment of the stage III and stage IV colon cancer. In the present study, intensity-modulated radiation therapy (IMRT) was performed with 6–8 MV X-ray. The adjuvant chemotherapy was either one of FOLFOX, XELOX or Capecitabine alone with median cycles of 2 (range from 2 to 6 cycles [22].

Patients with rectal cancer, as well as patients with the ascertained MSI status were excluded. The whole cohort was divided into two cohorts, with 923 patients in the training cohort from the January of 2004 to the November of 2013 and the other 923 patients in the validation cohort from December 2013 to the August 2015. Ethical approval was obtained from the institutions through the respective institutional review boards. The study protocol was designed in accordance with the guidelines outlined in the Declaration of Helsinki and was approved by the Ethics Committee of Sun Yat-sen University Cancer Center.

A standardized data collection form was designed to retrieve all relevant sociodemographic data (age, gender, pathologic subtype); preoperative baseline laboratory data: carcino-embryonic antigen (CEA), Carbohydrate antigen (CA199), albumin (ALB), C-reactive protein (CRP), etc.; staging data. All patients had received standard chemotherapies of FOLFIRI19 (47.2%), FOLFOX20 (33.5%), or XELOX21 (19.3%), and/or in combination with bevacizumab every 3 weeks.

Colon cancers were identified by ICD-O-3 site codes. If the cancer located in cecum, ascending colon, hepatic flexure of colon, and transverse colon, it would be defined as RCC, while those located in splenic flexure of colon, descending colon, sigmoid colon, and rectosigmoid were defined as LCC [13, 23–28]. Clinical stage was reclassified according to the criteria of the American Joint Commission on Cancer/International Union Against Cancer (AJCC/UICC). Overall survival (OS) was defined as the time from the date of primary treatment to the date of death from any cause or until the date of the last follow-up and the deadline of the follow-up was November 2016.

### Assessment of the CEA, CA199 and CRP

All samples were collected before any treatment and were tested within 24 h after collection. The supernatants were processed for analyzing CEA, CA199 on UniCelDxI 800 immunoassay system (Beckman Coulter, Brea, CA).Plasma CRP was measured using a high sensitivity assay (Beckman-Coulter, Woerden, The Netherlands) as described previously [29].

### MMR status determination

Immunohistochemistry was performed to examine the four most common mismatch repair proteins under the standard Envision two-step procedure. In brief, the slides were backed at 60°Cfor 2 h, cleared through xylene, rehydrated, then pre-treated in EDTA antigen retrieval buffer, treated with 3% hydrogen for 20 min to block endogenous peroxidase activities and then incubated with 10% normal goat serum at room temperature to block non-specific activity. Then, the slides were incubated overnight at 4°Cusing the following polyclonal antibodies, MLH1 (1:50; Beijing Zhong Shan -Golden Bridge Biological Technology, Beijing, China), PMS2 (1:50; Beijing Zhong Shan -Golden Bridge Biological Technology, Beijing, China), MSH2 (1:50; Beijing Zhong Shan -Golden Bridge Biological Technology, Beijing, China) and MSH6 (1:50; Beijing Zhong Shan -Golden Bridge Biological Technology, Beijing, China). After washing, the tissues were incubated with a secondary antibody (Envision; Dako, Glostrup, Denmark) for 1 h at room temperature. Finally, the sections were counterstained with 10% Mayer’s hematoxylin, dehydrated and mounted in Crystal Mount. Non-neoplastic colonic mucosa, stromal cells, infiltrating lymphocytes or the centers of lymphoid follicles were accepted as internal positive control and the known MMR deficient colorectal carcinomas used as external negative controls. Immunostaining was scored by two experienced pathologists and without any prior knowledge of the patients’ clinical data. Nuclear staining within tumor cells was defined as the normal expression, while complete absence of nuclear staining within tumor cells with concurrent internal positive controls was illustrated as negative protein expression. MLH1/PMS2/MSH2/MSH6 protein expression negative was defined as tumor with loss of MLH1/PMS2/MSH2/MSH6 protein visualized by light microscopy. Whatever one of these MLH1/PMS2/MSH2/MSH6 protein expressions is negative; it was defined as DMMR cohort. If the all the four protein is positive, the specimen then will de classified to the PMMR cohort.

### Statistical analysis

Continuous variables were expressed as mean ± standard deviation, median and range, and were transformed into dichotomous variables at median value. The threshold of CEA and C19–9 were established at 5 ng/ml and 37 U/ml as commonly suggested [30, 31]. Comparisons were performed using univariate logistic regression for categorical/continuous variable. Variables achieving significance at the level of *P* < 0.05 were entered into multivariate logistic regression analyses via stepwise procedures. Statistical data analyses were performed using SPSS 22.0 (SPSS, Chicago, IL, USA).

The PNI was calculated as10 × serum albumin value (g/dl) + 0.005 × peripheral lymphocyte count (per mm^3^). The optimal cutoff level for the neutrophil to lymphocyte ratio (NLR), platelet to lymphocyte ratio (PLR), CAR (C-reactive/Albumin Ratio)and PNI was determined using the median value [32]. The modified Glasgow Prognostic Score (mGPS) was entered into the analysis as categorical variables as descried before [33].

Kaplan–Meier method was used to calculate the OS survival curves, and difference was evaluated by the log-rank test. We also attempted to demonstrate the difference of PLR variation with the standard TNM classification between RCC and LCC using Nonparametric Test. all data has been deposited at Sun Yat-sen University Cancer Center for future reference (number RDDA2017000361).

## Results

### Patient characteristics and survival

A total of 1846 patients were included in the analyses for the analysis, with 744 patients in the RCC cohort and 1102 patients in the LCC. MSI status was successfully determined in 1846 patients. One thousand ninety-nine patients had received the chemotherapy and 378 patients had received radiotherapy. Patients in the training cohort were 923 patients and the other 923 patients were included in the validation cohort. The median follow-up time was for OS was 37 months (range: 4–138 months) in the whole cohort. Five-year OS was 86%in the whole cohort, 85.9% in LCC cohort and 88.7% RCC cohort, with the apparent poorer survival in the LCC (*P* = 0.003, HR = 1.475, 95% CI, 1.137–1.914), which is consistent with the previous study [34–37]. The patients’ characteristics plan to compare between RCC and LCC were summarized in Tables 1.

**Table 1:** Clinical and laboratory characteristics of the CRC, the RCC and the LCC

Characteristic	ALL			Training Cohort		Validation cohort	
	Number (%)			Number (%)		Number (%)	
	ALL	RCC	LCC	RCC	LCC	RCC	LCC
Age, years							
< 59	972 (52.7%)	427 (43.9%)	545 (56.1%)	220 (44.1%)	279 (55.9%)	207 (43.8%)	266 (56.2%)
≥ 59	874 (47.3%)	317 (36.3%)	557 (63.7%)	162 (38.2%)	262 (61.8%)	155 (34.4%)	295 (65.6%)
Sex							
Male	1106 (59.9%)	431 (39.0%)	675 (61.0%)	227 (39.6%)	346 (60.4%)	204 (38.3%)	329 (61.7%)
Female	740 (40.1%)	313 (42.3%)	427 (57.7%)	155 (44.3%)	195 (55.7%)	158 (40.5%)	232 (59.5%)
CRP, mg/L							
< 3.26	923 (50.0%)	312 (33.8%)	611 (66.2%)	160 (37.1%)	271 (62.9%)	152 (30.9%	340 (69.1%)
≥ 3.26	923 (50.0%)	432 (46.8%)	491 (53.2%)	222 (45.1%)	270 (54.9%)	210 (48.7%)	221 (51.3%)
WBCs, ×10^9^							
< 6.4	934 (50.6%)	363 (38.9%)	571 (61.1%)	178 (39.2%)	276 (60.8%)	185 (38.5%	295 (61.5%)
≥ 6.4	912 (49.4%)	381 (41.8%)	531 (58.2%)	204 (43.5%)	265 (56.5%)	177 (40.0%)	266 (60.0%)
Neutrophils, ×10^9^							
< 3.9	928 (50.3%)	349 (37.6%)	579 (62.4%)	178 (40.4%)	263 (48.6%)	171 (35.1%)	316 (64.9%)
≥ 3.9	918 (49.7%	395 (43.0%)	523 (57.0%)	204 (42.3%)	278 (57.7%)	191 (43.8%)	245 (56.2%)
Platelets, ×10^9^							
< 252	933 (50.5%)	294 (31.5%)	639 (68.5%)	157 (33.2%)	316 (66.8%)	137 (29.8%)	323 (70.2%)
≥ 252	913 (49.5%)	450 (49.3%)	463 (50.7%)	225 (50.0%)	225 (50.0%)	225 (48.6%)	238 (51.4%)
ALB, g/L							
< 40.5	924 (50.1%)	431 (46.6%)	493 (53.4%)	217 (45.4%)	261 (54.6%)	214 (48.0%)	232 (52.0%)
≥ 40.5	922 (49.9%)	313 (33.9%)	609 (66.1%)	165 (37.1%)	280 (62.9%)	148 (31.0%)	329 (69.0%)
CEA, ng/mL							
< 5	1066 (57.7%)	443 (41.6%)	623 (58.4%)	270 (40.8%)	392 (59.2%)	218 (37.8%)	359 (62.2%)
≥ 5	780 (42.3%)	301 (38.6%)	479 (61.4%)	112 (42.9%)	149 (57.1%)	144 (41.6%)	202 (58.4%)
CA199, U/mL							
< 27	1361 (73.7%)	536 (39.4%)	825 (60.6%)	270 (40.8%)	392 (59.2%)	266 (38.1%)	433 (61.9%)
≥ 27	485 (27.3%)	208 (42.9%)	277 (57.1%)	112 (42.9%)	149 (57.1%)	96 (42.9%)	128 (57.1%)
T lymphocytes, ×10^9^							
< 1.6	939 (50.9%)	399 (42.5%)	540 (57.5%)	202 (42.7%)	271 (57.3%)	197 (42.3%)	269 (57.7%)
≥ 1.6	907 (49.1%)	345 (38.0%)	562 (62.0%)	180 (40.0%)	270 (60.0%)	165 (36.1%)	292 (63.9%)
Monocytes, ×10^9^							
< 0.4	951 (51.5%)	382 (40.2%)	569 (59.8%)	206 (41.5%)	299 (58.5%)	176 (39.7%)	279 (61.3%)
≥ 0.4	895 (48.5%)	362 (40.4%)	533 (59.6%)	176 (41.2%)	251 (58.8%)	186 (39.7%)	282 (60.3%)
MMR							
D-MMR	1613 (87.4%)	588 (36.5%)	1025 (63.5%)	93 (66.0%)	48 (34.0%)	63 (68.5%)	29 (31.5%)
P-MMR	233 (12.6%)	156 (67.0%)	77 (33.0%)	289 (37.0%)	493 (63.0%)	299 (36.0%)	532 (64.0%)
TNM category							
Chemotherapy							
No	231 (12.5%)	101 (10.6%)	130 (14.5%)	71 (12.7%)	40 (10.9%)	60 (11.8%)	60 (14.5%)
Yes	1499 (81.2%)	799 (84.1%)	700 (78.1%)	429 (77.0%)	300 (82.0%)	430 (84.3%)	340 (82.3%)
Unknown	116 (6.3%)	50 (5.3%)	66 (7.4%)	57 (10.2%)	26 (7.1%)	20 (3.9%)	13 (3.1%)
Radiotherapy							
No	1322 (71.6%)	611 (70.1%)	711 (73.0%)	411 (74.5%)	250 (67.4%)	355 (65.7%)	306 (79.9%)
Yes	378 (20.5%)	191 (21.9%)	187 (19.2%)	111 (20.1%)	78 (21.0%)	145 (26.9%)	44 (11.5%)
Unknown	146 (7.9%)	70 (8.0%)	76 (7.8%)	30 (5.4%)	43 (11.6%)	40 (7.4%)	33 (8.6%)
TNM category							
1	168 (9.1%)	34 (20.2%)	134 (79.8%)	15 (20.8%)	57 (79.2%)	19 (19.8%)	77 (80.2%)
2	839 (45.4%)	376 (44.8%)	463 (55.2%)	176 (47.8%)	192 (52.2%)	200 (42.5%)	271 (57.5%)
3	518 (28.1%)	202 (39.0%)	316 (61.0%)	100 (39.8%)	151 (60.2%)	102 (38.2%)	165 (61.8%)
4	321 (17.4%)	132 (41.1%)	189 (58.9%)	91 (39.2%)	141 (60.8%)	41 (46.1%)	48 (53.9%)
PLR		744	1102				
< 154.96	923 (50.0%)	295 (32.0%)	628 (68.0%)	160 (34.2%)	308 (65.8%	135 (29.7%)	320 (70.3%)
≥ 154.96	923 (50.0%)	449 (48.6%)	474 (51.4%)	222 (48.8%)	233 (51.2%)	227 (48.5%)	241 (51.5%)
NLR							
< 2.35	923 (50.0%)	330 (35.8%)	593 (64.2%)	165 (37.1%)	280 (62.9%)	165 (34.5%)	313 (65.5%)
≥ 2.35	923 (50.0%)	414 (44.9%)	509 (55.1%)	217 (45.4%)	261 (54.6%)	197 (44.3%)	248 (55.7%)
PNI		744	1102				
< 48.88	921 (49.9%)	363 (39.4%)	558 (60.6%)	182 (40.1%)	272 (59.9%)	181 (38.8%)	286 (61.2%)
≥ 48.88	925 (50.1%	381 (41.2%)	544 (58.8%)	200 (42.6%)	269 (57.4%)	181 (39.7%)	275 (60.3%)
CAR		744	1102				
< 0.08	923 (50.0%)	306 (33.2%)	617 (66.8%)	157 (36.3%)	275 (63.7%)	149 (30.3%)	342 (69.7%)
≥ 0.08	923 (50.0%)	438 (47.5%)	485 (52.5%)	225 (45.8%)	266 (54.2%)	213 (49.3%)	219 (50.7%)
mGPS							
0	1243 (67.3%)	441 (35.5%)	802 (64.5%)	223 (37.5%)	371 (62.5%)	218 (33.6%)	431 (66.4%)
1	406 (22.0%)	200 (49.3%)	206 (50.7%)	114 (50.2%)	113 (49.8%)	86 (48.0%)	93 (52.0%)
2	197 (10.7%)	103 (52.3%)	94 (47.7%)	45 (44.1%)	57 (55.9%	58 (61.1%)	37 (38.9%)
Survival status							
Live	1586 (85.9%)	660 (41.6%)	926 (58.4%)	316 (44.4%)	396 (55.6%)	344 (39.4%)	530 (60.6%)
Dead	260 (14.1%)	84 (32.3%)	176 (67.7%)	66 (31.3%)	145 (68.7%)	18 (36.7%)	31 (63.3%)

*Abbreviations*: *CRP* C-reactive protein, *WBCs* White blood cells, *ALB* Albumin, *CA199* Carbohydrate Atigen 19–9, *CEA* Carcinoembryonic antigen, *MMR* Mismatch repair; *PLR* The platelet to lymphocyte ratio, *NLR* The neutrophil to lymphocyte ratio, *PNI* 10 × serum albumin value (g/dl) + 0.005 × peripheral lymphocyte count (per mm3), *mGPS*, Glasgow Prognostic Score incorporates raised circulating C-reactive protein (CRP) and hypoalbuminemia; Undifferentiated, undifferentiated non-keratinizing carcinoma; Differentiated, differentiated carcinoma

### Dfferent characteristics between RCC and LCC

Patients in the training cohort with left-sided colon cancer had early tumor stages, higher inflammatory index (CRP, pateletes, PLR, NLR, CAR, mGPS), higher tumor marker CEA, higher ALB and higher probability of microsatelite stability in the univariate analysis. All significant variables were entered into multivariate logistic regression; MMR status (*P* < 0.001), PLT (*P* = 0.004), CEA (*P* < 0.001), PLR (*P* = 0.011), TNM stage (*P* = 0.001) retained independent prognostic significance for the location of CRC. Detailed summaries of the multivariate analyses are shown in Tables 2. All the charateristics were anlylzed in validation cohort, MMR status (*P* < 0.001), age (*P* = 0.007), ALB (*P* < 0.001), PLR (*P* = 0.022) and TNM stage (*P* = 0.011) were proven to be independent different prognostic factors (Table 3). Obviously, MMR status, PLR, TNM stage were the significant difference demonstrated in both cohorts and PLR was the merely significant different inflammotory factor between the LCC and the RCC.

**Table 2:** Associations between the clinical and laboratory characteristics of the patients and location of CRC in univariate and multivariate logistic regression analysis in the training cohort

Characteristic	Univariate			Multivariate		
	HR	95% CI	*P*-value	HR	95% CI	*P*-value
Age (< 59 years vs. ≥ 59)	1.275	0.980–1.660	0.071			
Gender (Male vs. Female)	1.212	0.926–1.586	0.162			
CRP, mg/L (≥ 3.26 vs. < 3.26)	1.393	1.069–1.813	0.014			0.835
WBCs, ×10^9^ (≥ 6.4 vs. < 6.4)	1.194	0.918–1.552	0.186			
Neutrophils, ×10^9^ (≥ 3.9 vs. < 3.9)	1.084	0.834–1.410	0.546			
Platelets, ×10^9^ (≥ 252 vs. < 252)	2.013	1.543–2.626	<0.001	1.563	1.151–2.124	0.004
ALB, g/L (≥ 40.5 vs. < 40.5)	0.709	0.545–0.922	0.010			0.275
CA199, U/mL (≥ 27 vs. < 27)	1.091	0.817–1.458	0.555			0.635
CEA, ng/mL (≥ 5 vs. < 5)	1.504	1.154–1.959	0.003	1.819	1.350–2.450	<0.001
T lymphocytes, ×10^9^ (≥1.6vs. < 1.6)	0.894	0.688–1.163	0.404			
Monocytes, ×10^9^ (≥ 0.4 vs. < 0.4)	0.987	0.759–1.284	0.923			
MMR, (P vs. D)	0.303	0.207–0.441	<0.001	0.309	0.209–0.457	<0.001
Chemotherapy						
No	1.220	0.911–1.634	0.182			
Yes	1.252	0.932–1.681	0.135			
0.237 Unknown	1.026	0.767–1.372	0.862			
Radiotherapy						
No	1.234	0.920–1.655	0.160			
Yes	1.235	0.871–1.750	0.237			
Unknown	0.975	0.678–1.401	0.889			
TNM			<0.001			0.001
1	1.000	1.000		1.000	1.000	
2	3.483	1.903–6.375	<0.001	3.126	1.665–5.869	<0.001
3	2.517	1.351–4.689	0.004	2.470	1.291–4.725	0.006
4	2.452	1.310–4.590	0.005	2.345	1.217–4.516	0.011
PLR	1.834	1.407–2.391	<0.001	1.454	1.071–1.975	1.4540.022d
NLR	1.411	1.084–1.836	0.010			0.443
CAR	1.482	1.137–1.930	0.004			0.428
PNI	1.111	0.855–1.444	0.431			
mGPS			0.004			0.428
0	1.000	1.000				
1	1.678	1.233–2.285	0.001			0.195
2	1.313	0.859–2.008	0.208			0.850

*Abbreviations*: *CRP* C-reactive protein, *WBCs* White blood cells, *ALB* Albumin, *CA199* Carbohydrate Atigen 19–9, *CEA* Carcinoembryonic antigen, *MMR* Mismatch repair, *PLR* The platelet to lymphocyte ratio, *NLR* The neutrophil to lymphocyte ratio; PNI, 10 × serum albumin value (g/dl) + 0.005 × peripheral lymphocyte count (per mm3), *mGPS* Glasgow Prognostic Score incorporates raised circulating C-reactive protein (CRP) and hypoalbuminemia; Undifferentiated, undifferentiated non-keratinizing carcinoma; Differentiated, differentiated carcinoma

**Table 3:** Associations between the clinical and laboratory characteristics of the patients and location of CRC in univariate and multivariate logistic regression analysis in the validation cohort

Characteristic	Univariate			Multivariate		
	HR	95% CI	*P*-value	HR	95% CI	*P*-value
Age (< 59 years vs. ≥ 59)	1.481	1.135–1.933	0.004	1.384	1.036–1.848	0.028
Gender (Male vs. Female)	1.098	0.841–1.435	0.491			
CRP, mg/L (≥ 3.26 vs. < 3.26)	2.126	1.625–2.781	<0.001			
WBCs, ×10^9^ (≥ 6.4 vs. < 6.4)	1.061	0.815–1.382	0.660			
Neutrophils, ×10^9^ (≥ 3.9 vs. < 3.9)	1.441	1.105–1.878	0.007			
Platelets, ×10^9^ (≥ 252 vs. < 252)	1.545	1.125–2.121	0.007			
ALB, g/L (≥ 40.5 vs. < 40.5)	0.488	0.373–0.638	<0.001	0.532	0.398–0.710	<0.001
CA199, U/mL (≥ 27 vs. < 27)	1.221	0.899–1.657	0.200			
CEA, ng/mL (≥ 5 vs. < 5)	1.174	0.894–1.541	0.248			
T lymphocytes, ×10^9^ (≥1.6vs. < 1.6)	0.772	0.592–1.006	0.055			
Monocytes, ×10^9^ (≥ 0.4 vs. < 0.4)	1.046	0.803–1.362	0.741			
MMR, (P vs. D)	0.259	0.163–0.411	<0.001	0.317	0.195–0.515	<0.001
TNM			<0.001			0.011
1	1.000	1.000		1.000	1.000	
2	2.991	1.753–5.103	<0.001	2.365	1.357–4.123	0.002
3	2.505	1.432–4.384	0.001	2.175	1.215–3.892	0.009
4	3.462	1.803–6.648	<0.001	2.963	1.501–5.850	0.002
PLR	2.233	1.703–2.927	<0.001	1.451	1.056–1.993	0.022
NLR	1.507	1.155–1.965	0.002			
CAR	2.232	1.705–2.923	<0.001			
PNI	1.040	0.798–1.355	0.771			
mGPS			<0.001			
0	1.000	1.000				
1	3.099	1.989–4.828	<0.001			
2	1.695	1.022–2.812	0.041			

*Abbreviations*: *CRP* C-reactive protein, *WBCs* White blood cells, *ALB* Albumin, *CA199* Carbohydrate Atigen 19–9, *CEA* Carcinoembryonic antigen, *MMR* Mismatch repair, *PLR* The platelet to lymphocyte ratio, *NLR* The neutrophil to lymphocyte ratio; PNI, 10 × serum albumin value (g/dl) + 0.005 × peripheral lymphocyte count (per mm3), *mGPS* Glasgow Prognostic Score incorporates raised circulating C-reactive protein (CRP) and hypoalbuminemia; Undifferentiated, undifferentiated non-keratinizing carcinoma; Differentiated, differentiated carcinoma

### PLR and survival

**Fig. 1:**
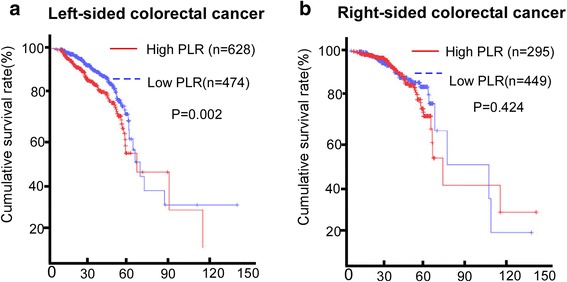
Prognostic value of the PLR (platelet and neutrophils) for OS. **a** The whole cohort, **b** the RCC cohort, and (**c**) the LCC cohort

PLR had the ability to distinguish patients had poorer survival in the LCC cohort by log-rank test (*P* = 0.002, HR = 0.1.261, 95%CI, 1.087–1.462) (Fig. 1a). However, the better survival of the lower PLR was not observed in the RCC cohort (*P* = 0.424, HR = 1.094, 95% CI, 0.877–1.365, Fig. 1b). The PLR prognostic value merely exists in the early-staged TNM staging but not the advanced stage (Additional file 1: Figure S1A and B). Additionally, the higher PLR have poorer survival than the lower PLR in the LCC cohort (*P* = 0.002) but not the RCC (*P* = 0.869) in the early-staged TNM staging (Additional file 1: Figure S1 C and D).

### PLR variation with TNM staging

**Fig. 2:**
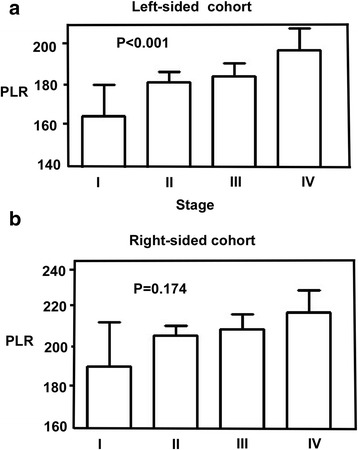
Variation of the continuous variable PLR (platelet and neutrophils) with TNM stage. **a** The whole cohort, **b** the RCC cohort, and (**c**) the LCC cohort

The variation trend between systemic inflammatory factors and the tumor staging was shown in Fig. 2. We found that there were significant interactions between tumor stages (I to IV) with PLR in LCC cohort (*P* < 0.001, Fig. 2a), with the lowest values in stage I and the highest in stage IV. However, the relationship did not sustain in subgroup RCC patients, as shown in (*P* = 0.174, Fig. 2b).

## Discussion

Currently, most studies had focused on the biology, microenvironment and survival difference in RCC and LCC, however, there is still no report regarding the inflammatory distinction between them [37–41].To our knowledge this is the first population-based research exploring the inflammatory-related index disparity of tumor location in CRC.

As shown in Table 1, the clinical characteristics such as TNM staging, MMR status, were significantly differ between RCC and LCC in training cohort and age, TNM staging, MMR status were different in the validation cohort. Additionally, our results showed that CRP, PLT, ALB, PLR NLR, CAR, mGPS were the different inflammatory factor between the LCC and the RCC in the training cohort. Similarly, CRP, PLT, neutrophils, ALB, PLR, NLR, CAR, mGPS were the different inflammatory index between the LCC and the RCC in the validation cohort. However, PLR was the only inflammatory index among CEA CRP, neutrophils, platelets, ALB, PLR, NLR, CAR, and mGPS that differed significantly between RCC and LCC in multivariate analysis verified in both cohorts. The other independent factors in the training cohort were PLT, CEA, MMR status, and TNM stage and age, ALB, MMR and TNM stage were the independent factor in the validation cohort. Together, our results showed that PLR might be a vital different inflammatory factor between RCC and LCC. Among these five inflammatory factors, PLR, NLR, mGPS, CAR, PNI, why is only the PLR indicating the difference between the LCC and RCC, the reasons still unknown. As the previous studies shown, that the OS or DFS (disease-free survival) prognostic value were indeed validated for these five factors in CRC [42–46]. However, there is no report regarding the difference of their prognostic value for in the LCC and RCC. We assume that other systemic inflammatory response parameters (such as NLR, PNI, mGPS, CRP, CAR) can not represent the LCC and RCC inflammatory difference is that hypoalbuminemia reflects a malnutrition but not inflammatory reaction [47] and that is why the CAR, mGPS and PNI were not the representative index between the LCC and the RCC. Although the clinical significance of NLR is still unclear, it has been pointed out that this parameter may transferred between the pro-inflammatory response (i.e. high value of neutrophils and low value of lymphocytes) and an immune pattern (i.e. low value of neutrophils and high value of lymphocytes) [42].

The PLR has been demonstrated as a prognostic factor in several malignant tumors, including colorectal cancer, gastric cancer, esophageal carcinoma, esophageal squamous cell carcinoma (ESCC), small cell lung cancer [48–52]. The role of both platelets and lymphocyte as independent regulators of various processes in cancer has been known for long. However, the exact mechanism of the inflammatory index difference in the LCC and RCC has not been illustrated although *Gervaz* et al. reported that CRC is a heterogeneous disease and could be differentiated into two anatomical and functional entities [53].

Interestingly, the difference of PLR in our study has significantly translated into OS difference only in LCC rather than RCC, and PLR changes with TNM stage only in LCC. So, what is the inner link between PLR and LCC? According to *Guinney’s* (14) research, LCC related to CMS2, which characterized as epithelial, chromosomally unstable, marked WNT and MYC signaling activation; RCC related to CMS1, which characterized as hypermutated, microsatellite unstable, strong immune activation. Obviously, LCC related to inflammation, but RCC not, so, it is consistent with the opinion that the PLR reflect inflammation too. *Chapman* et al. has demonstrated that platelets present antigen to T cells in a platelet MHCI (major histocompatibility complex I) dependent manner, which indicate platelets not only support and promote acquired immune responses, but may also directly participate in the initiation of acquired immune responses. *Liang* et al. has revealed that the over-activation of platelets enhances survival of tumor cells in circulation by the CD62P ligand [54]. It has also been convincingly demonstrated that platelet addition to tumor cells can impede natural killer cell mediated recognition and elimination of tumor cells, which may prime the tumor cells for metastasis [55]. Platelet could activate the epidermal growth factor receptor (EGFR) and downstream signals of DNA-dependent protein kinase (DNA-PK)-a ubiquitous DNA repair enzyme. Prior studies have shown that the formation of the EGFR: DNA-PK complex could maintain DNA repair [56, 57]. Therefore, we suppose that the activated platelets not only promote CTCs to survive, but also enhance metastasis ability of tumor cells directly, especially in the LCC [58, 59]. Platelets’ role as inducers of intravascular NETosis (neutrophil extracellular traps) has also been revealed with the effect to promote thrombosis, systemic inflammation, and relapse of the tumor disease [60–62]. Beyond the routine role as chief effector cells in hemostasis and thrombosis, platelets also play a vital role as inflammatory cells since its activation is crucial for the metastasis CTCs cells to escape from immune cells attack for adapting the blood microenvironment. In sum, the activated platelets may were used as stimulator in the tumor progression and may accelerate early cancer development [63, 64].

Finally, with the increase of TNM stage, PLR significantly increased either in LCC rather than RCC in our study. The LCC exhibit the characteristics of higher rates of microsatellite stability (MSS) [65] and a notable feature of PMMR. Concerning the clinical relevance, an inability to respond to adverse environmental stressors might have clear implications for the success of chemotherapy in these tumors. It has recently been shown that MSS tumors show a good response to chemotherapy, but those patients population with increase of PLR which represent the inflammation reaction, so, targeted inflammation or platelet may be the direction of treatment for those patients.

The study was conducted retrospectively and selection bias may exist. However, we included a relatively large cohort to assess the difference of PLR in LCC and RCC in independent training cohort and validation cohort. Of course, additional validation of the PLR is necessary in prospective datasets. In summary, this study suggests that the prognostic value of the PLR, a continuous variable, may help to stratify LCC and RCC patient and guide treatment especially in the LCC.

## Conclusion

This is the first study that regarding the inflammatory status between the LCC and the RCC and we found the PLR was the merely different inflammatory parameter between the LCC and the RCC. Additionally, the PLR variation trend with the tumor staging was shown in only in the LCC.

## Additional file

Additional file 1: Figure S1. The prognostic value in the stratified TNM staging (A, staging I + II + III; B, staging IV); The prognostic value in the LCC (C) and the RCC (D) in the staging I + II + III, respectively. (TIFF 13481 kb)

## Abbreviations

ALB: Albumin
CA199: Carbohydrate antigen
CEA: Carcino-embryonic antigen
CMS: Consensus molecular subtypes
CRC: Colon cancer
CRP: C-reactive protein
d-MMR: Mismatch repair deficiency
DNA-PK: DNA-dependent protein kinase
ESCC: Esophageal squamous cell carcinoma
LCC: Left-sided colon cancer
mGPS: Glasgow prognostic score
MHCI: Major histocompatibility complex I
MSI-high: Microsatellite instability-high
MSS: Microsatellite stability
NETosis: Neutrophil extracellular traps
NK: Natural killer cell
NLR: Neutrophil lymphocyte ratio
OS: Overall survival
PFS: Progression-free survival
PLR: Platelet lymphocyte ratio
P-MMR: Mismatch repair–proficient
p-MMR: Mismatch-repair proficient
PNI: Nutritional Index
RCC: Right-sided colon cancer
