# Supplementary figures and images for: Clinical baseline and prognostic difference of platelet lymphocyte ratio (PLR) in right-sided and let-sided colon cancers
Source: BMC Cancer. 2017 Dec 20;17:873. doi: 10.1186/s12885-017-3862-8 (PMC5738180; doi:10.1186/s12885-017-3862-8)

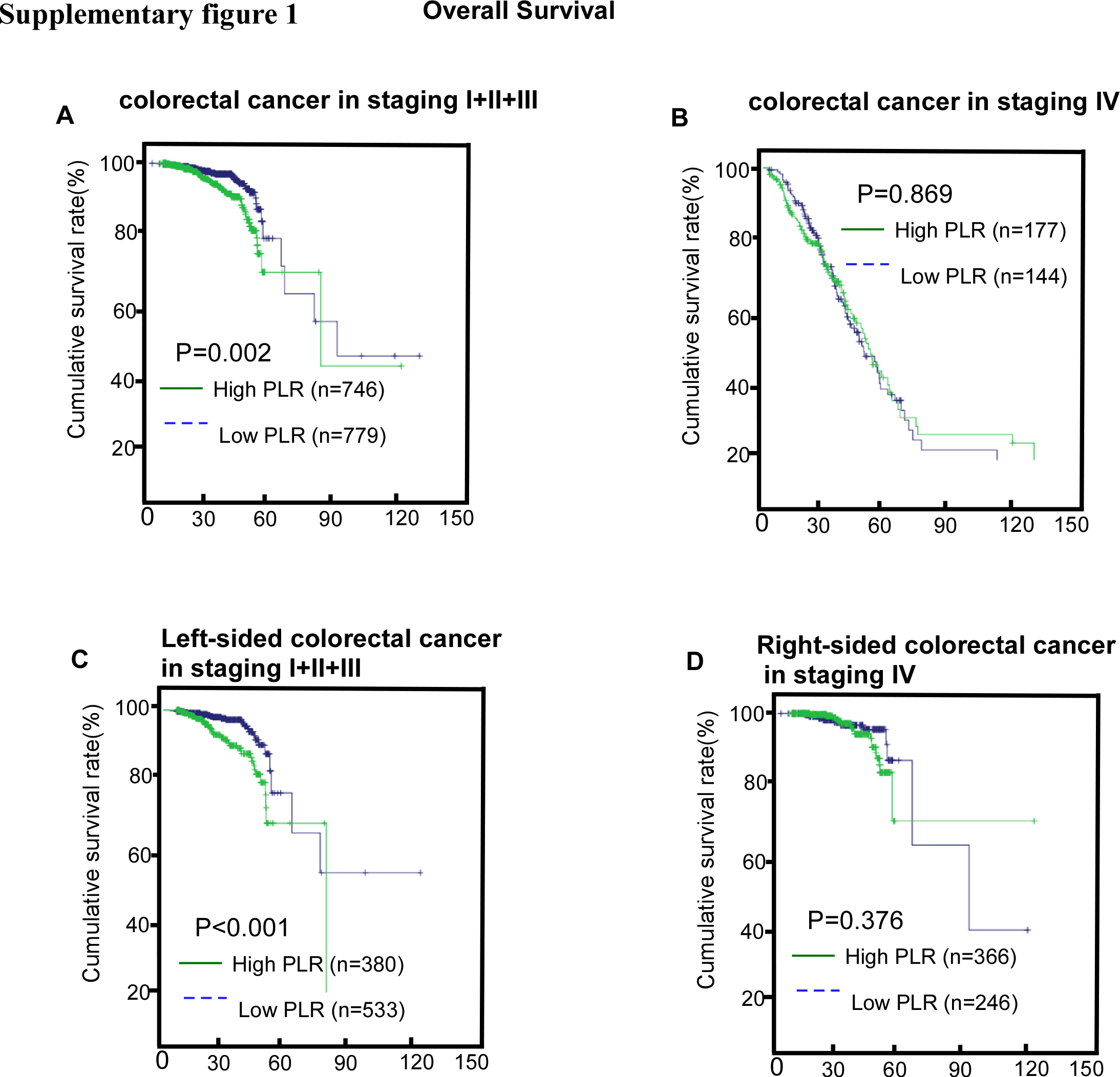

Supplement: Additional file 1: Figure S1. — The prognostic value in the stratified TNM staging (A, staging I + II + III; B, staging IV); The prognostic value in the LCC (C) and the RCC (D) in the staging I + II + III, respectively. (TIFF 13481 kb) [file 12885_2017_3862_MOESM1_ESM.tif]
